# Defective Influenza A Virus RNA Products Mediate MAVS-Dependent Upregulation of Human Leukocyte Antigen Class I Proteins

**DOI:** 10.1128/JVI.00165-20

**Published:** 2020-06-16

**Authors:** Mir Munir A. Rahim, Brendon D. Parsons, Emma L. Price, Patrick D. Slaine, Becca L. Chilvers, Gregory S. Seaton, Andrew Wight, Daniel Medina-Luna, Sayanti Dey, Shannen L. Grandy, Lauryn E. Anderson, Natalia Zamorano Cuervo, Nathalie Grandvaux, Marta M. Gaglia, Alyson A. Kelvin, Denys A. Khaperskyy, Craig McCormick, Andrew P. Makrigiannis

**Affiliations:** a Department of Biomedical Sciences, University of Windsor, Windsor, Ontario, Canada; b Department of Microbiology & Immunology, Dalhousie University, Halifax, Nova Scotia, Canada; c Department of Biochemistry and Molecular Biology, Dalhousie University, Halifax, Nova Scotia, Canada; d Department of Cancer Immunology and Virology, Dana Farber Cancer Institute, Boston, Massachusetts, USA; e Centre Hospitalier de l’Université de Montréal (CRCHUM), Montréal, Quebec, Canada; f Department of Biochemistry and Molecular Medicine, Faculty of Medicine, Université de Montréal, Montréal, Quebec, Canada; g Department of Molecular Biology and Microbiology, Tufts University School of Medicine, Boston, Massachusetts, USA; h Department of Pediatrics, Dalhousie University, Halifax, Nova Scotia, Canada; St. Jude Children's Research Hospital

**Keywords:** influenza A virus, NK cells, DI RNAs, mvRNAs, RIG-I, MAVS, class I MHC, class I HLA, KIR, interferon

## Abstract

Influenza A virus (IAV) increases the presentation of class I human leukocyte antigen (HLA) proteins that limit antiviral responses mediated by natural killer (NK) cells, but molecular mechanisms for these processes have not yet been fully elucidated. We observed that infection with A/Fort Monmouth/1/1947(H1N1) IAV significantly increased the presentation of HLA-B, -C, and -E on lung epithelial cells. Virus entry was not sufficient to induce HLA upregulation because UV-inactivated virus had no effect. Aberrant internally deleted viral RNAs (vRNAs) known as mini viral RNAs (mvRNAs) and defective interfering RNAs (DI RNAs) expressed from an IAV minireplicon were sufficient for inducing HLA upregulation. These defective RNAs bind to retinoic acid-inducible gene I (RIG-I) and initiate mitochondrial antiviral signaling (MAVS) protein-dependent antiviral interferon (IFN) responses. Indeed, MAVS was required for HLA upregulation in response to IAV infection or ectopic mvRNA/DI RNA expression. The effect was partially due to paracrine signaling, as we observed that IAV infection or mvRNA/DI RNA-expression stimulated production of IFN-β and IFN-λ1 and conditioned media from these cells elicited a modest increase in HLA surface levels in naive epithelial cells. HLA upregulation in response to aberrant viral RNAs could be prevented by the Janus kinase (JAK) inhibitor ruxolitinib. While HLA upregulation would seem to be advantageous to the virus, it is kept in check by the viral nonstructural 1 (NS1) protein; we determined that NS1 limits cell-intrinsic and paracrine mechanisms of HLA upregulation. Taken together, our findings indicate that aberrant IAV RNAs stimulate HLA presentation, which may aid viral evasion of innate immunity.

**IMPORTANCE** Human leukocyte antigens (HLAs) are cell surface proteins that regulate innate and adaptive immune responses to viral infection by engaging with receptors on immune cells. Many viruses have evolved ways to evade host immune responses by modulating HLA expression and/or processing. Here, we provide evidence that aberrant RNA products of influenza virus genome replication can trigger retinoic acid-inducible gene I (RIG-I)/mitochondrial antiviral signaling (MAVS)-dependent remodeling of the cell surface, increasing surface presentation of HLA proteins known to inhibit the activation of an immune cell known as a natural killer (NK) cell. While this HLA upregulation would seem to be advantageous to the virus, it is kept in check by the viral nonstructural 1 (NS1) protein, which limits RIG-I activation and interferon production by the infected cell.

## Abstract (precis)

Human leukocyte antigens (HLAs) are cell surface proteins that regulate innate and adaptive immune responses to viral infection by engaging with receptors on immune cells. Many viruses have evolved ways to evade host immune responses by modulating HLA expression and/or processing. Here, we provide evidence that aberrant RNA products of influenza virus genome replication can trigger retinoic acid-inducible gene I (RIG-I)/mitochondrial antiviral signaling (MAVS)-dependent remodeling of the cell surface, increasing surface presentation of HLA proteins known to inhibit the activation of an immune cell known as a natural killer (NK) cell. While this HLA upregulation would seem to be advantageous to the virus, it is kept in check by the viral nonstructural 1 (NS1) protein, which limits RIG-I activation and interferon production by the infected cell.

## INTRODUCTION

Influenza A viruses (IAVs) infect human airway epithelial cells and trigger innate host defenses that limit virus replication and spread (1). Human respiratory epithelial cells are equipped with pattern recognition receptors (PRRs), including Toll-like receptors (TLRs) and retinoic acid-inducible gene I (RIG-I)-like receptors (RLRs) that bind viral RNA and transduce signals to initiate the production of interferons (IFNs) and proinflammatory cytokines. In endosomes, TLR3 binds double-stranded (ds) viral RNAs (vRNAs) and initiates a signaling cascade to activate the proinflammatory transcription factor NF-κB (2). However, during IAV infection of epithelial cells, the bulk of vRNA ligands for PRRs are located in the nucleus and cytoplasm; here, RIG-I serves as the chief sensor for IAV RNA species that include panhandle structures generated by complementary base pairing of 5′ and 3′ ends of vRNAs (3–8). Following vRNA binding, RIG-I associates with the mitochondrial antiviral signaling (MAVS) adaptor protein on the surface of mitochondria (9) and peroxisomes (10, 11); subsequent MAVS oligomerization causes recruitment and activation of interferon regulator factor 3 (IRF3) and IRF7 and transcription of antiviral type I IFNs (IFN-α and IFN-β) and type III IFNs (IFN-λ1 to 3).

Both *in vitro* and *in vivo* studies have shown that during viral RNA transcription and replication, IAVs generate defective RNA products missing portions of the viral RNAs (12). These include defective interfering (DI) RNAs, which are ≥178-nucleotide (nt)-long subgenomic RNAs that can be incorporated into defective viral particles (13); mini viral RNAs (mvRNA) that are similar in structure to DI RNAs but are considerably shorter (∼56 to 125 nt long) (14); and the 22- to 27-nt-long small viral RNA (svRNA) corresponding to the 5′ end of vRNA (15). Both DI RNAs and mvRNAs retain panhandle structures with closely apposed 5′ and 3′ ends that are ligands for RIG-I, which initiates antiviral signal transduction. Defective viral RNAs are thought to limit productive viral replication and the pathogenic effects of infection, in part, by being triggers for innate immune responses. mvRNAs are potent inducers of type I IFN production, whereas svRNAs fail to trigger IFN responses (14). However, it is unknown precisely how these defective viral RNAs affect the recognition of IAV-infected cells by the immune system.

Among the immune effector cells recruited to the lungs within days after IAV infection are natural killer (NK) cells, which possess cytotoxic function against virus-infected cells (16, 17). NK cells, whose function is regulated by an array of activating and inhibitory receptors, have an important role in the control of IAV infection in mice (18, 19). The activating NKp44 and NKp46, as well as costimulatory 2B4 and NTB-4, receptors aid in recognition and killing of IAV-infected cells by binding hemagglutinin (HA) protein on their surface (20–22). In mice, NKp46 deficiency results in increased morbidity and mortality following IAV infection, demonstrating the importance of this NK cell receptor in the control of infection (23, 24). Because binding of NKp46 to the viral HA protein is dependent on sialylation of the *O*-glycosylated residues of NKp46, IAV can counter this recognition by cleaving the receptor sialic acids using the viral neuraminidase (NA) (25, 26). IAV can also circumvent NK cell-mediated antiviral responses by increasing the expression of inhibitory ligands, such as the class I human leukocyte antigen (HLA), also known as the human major histocompatibility complex class I (MHC-I), on the surface of infected cells. Class I HLA molecules are recognized by the human killer cell immunoglobulin-like receptors (KIRs) on NK cells (27). Increasing the binding of inhibitory KIRs to class I HLA proteins on IAV-infected cells has been shown to inhibit NK cell function (28). Previously, we demonstrated that IAV infection in mice is associated with increased expression of mouse MHC-I on lung epithelial cells (29). On mouse NK cells, the functional analogues of KIRs are inhibitory Ly49 receptors; we observed that disruption of inhibitory Ly49:MHC-It interactions improved survival of IAV-infected mice. Our study demonstrated that upregulation of MHC-I helps IAV evade NK cell-mediated immune responses, but the mechanism by which MHC-I is upregulated during IAV infection is not fully understood.

NK cell receptors bind to cognate ligands on the surface of infected cells and integrate activating and inhibitory signals that dictate the extent of NK cell activation (30). Knowing this, we initiated the current study to better understand how IAV infection affects the expression of ligands for NK cell receptors on the surface of infected epithelial cells. In-depth bioinformatic analysis of publicly available gene expression data sets revealed that IAV infection modulates the expression of a wide array of NK cell ligands, most notably, class I HLA genes that were consistently upregulated across many *in vitro* infection studies that employed different IAV strains and epithelial cell models. We complemented these findings using an A549 lung epithelial cell infection model. We observed a significantly increased presentation of class I HLA and non-classical HLA-E on A/Fort Monmouth/1/1947(H1N1) IAV-infected A549 cells. We used IAV minireplicons and MAVS-knockout A549 cells to demonstrate that mvRNAs and DI RNAs are sufficient to increase HLA presentation in a MAVS-dependent manner. IAV infection or ectopic mvRNA/DI RNA-expression stimulated production of IFN-β and IFN-λ, and conditioned media from these cells elicited modest increases in HLA presentation from naive epithelial cells. Janus kinase (JAK) proteins transduce signals downstream from type I cytokine receptors and IFN receptors; using the Jak1/Jak2 inhibitor ruxolitinib (Rux), we demonstrated that Jak1 and/or Jak2 play major roles in HLA upregulation triggered by IAV replication intermediates. Finally, we determined that IAV NS1 limits cell-intrinsic and paracrine mechanisms of HLA upregulation. Collectively, our data indicate that aberrant IAV mvRNAs and DI RNAs stimulate HLA presentation, which may aid viral evasion of immune surveillance.

## RESULTS

### Influenza A virus infection alters cell surface expression of ligands for NK cell receptors.

**FIG 1:**
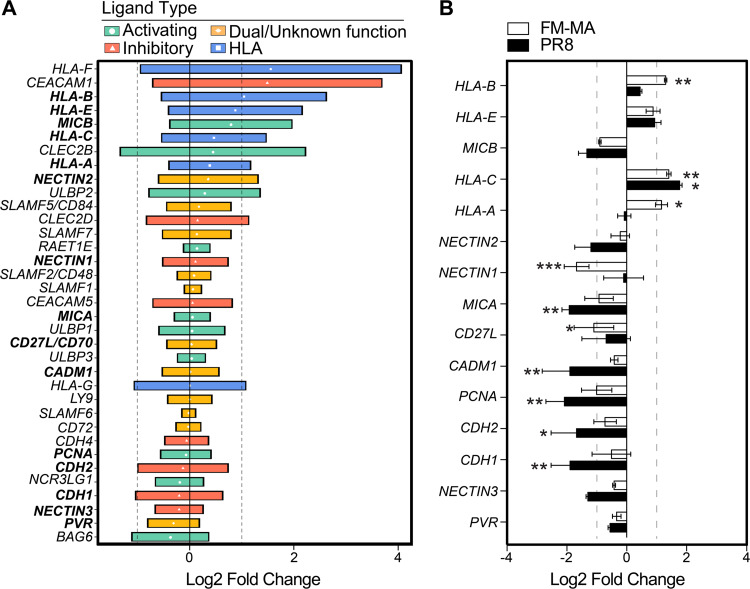
IAV infection of epithelial cells increases class I HLA gene expression. (A) Expression of NK cell ligands from 18 publicly available gene expression data sets from *in vitro* IAV infection of A549 cells and primary human lung cells. NK ligands are classified as activating (green), ambiguous function (orange), and inhibitory (red). Class I HLA proteins are indicated in blue. Data are presented as the log_2_ fold change relative to uninfected controls for each data set; median values with interquartile range (IQR) are shown. Vertical dashed lines indicate 2-fold change thresholds. (B) A549 cells were infected with PR8 or FM-MA or mock-infected for 17 h, and RNA was harvested for RT-qPCR. The relative expression of NK cell ligands was expressed as log_2_ fold change relative to mock-infected controls. Vertical dashed lines indicate 2-fold change thresholds. N = 3; *, *P* < 0.05; **, *P* < 0.01; ***, *P* < 0.001.

NK cells control immune responses to IAV infection *in vivo* (18). NK cell receptors bind to cognate ligands on the surface of infected cells and integrate activating and inhibitory signals that dictate the extent of NK cell engagement (30). We performed an in-depth bioinformatic analysis of publicly available gene expression data sets (see Table S1 in the supplemental material) to better understand how expression of known NK cell ligands is modulated by IAV infection *in vitro*. By focusing on data sets from *in vitro* IAV infections of standard epithelial cell models, including primary human lung epithelial cells and alveolar adenocarcinoma A549 cells, we learned that the expression of most known ligands for NK cell receptors is altered during IAV infection (Fig. 1A). We observed a consistent trend of upregulation of HLA transcripts in multiple epithelial cell lines in response to infection by diverse IAV strains. These included the HLA-A, -B, -C, and -E proteins that present peptides to immune cells and bind inhibitory receptors on NK cells, as well as HLA-F, which binds to KIR receptors with context-dependent activating and inhibitory properties.

To confirm reports of NK cell ligand modulation, we infected A549 cells with A/Puerto Rico/8/1934(H1N1) (PR8) at an MOI of 1 for 16 h, at which point RNA was harvested and analyzed by reverse transcriptase quantitative PCR (RT-qPCR), which revealed statistically significant increases in HLA-C and significant decreases in MICA, MICB, NECTIN3, CADM1, CDH1, CDH2, and PCNA in PR8-infected A549 cells (Fig. 1B). In contrast, infection of A549 cells with the mouse-adapted A/Fort Monmouth/1/1947(H1N1) (FM-MA) strain that we previously utilized to study NK cell responses to IAV infection in mice (29) caused significant increases in steady-state mRNA levels of HLA-A, -B, and -C, without causing statistically significant decreases in other NK cell ligands.

**FIG 2:**
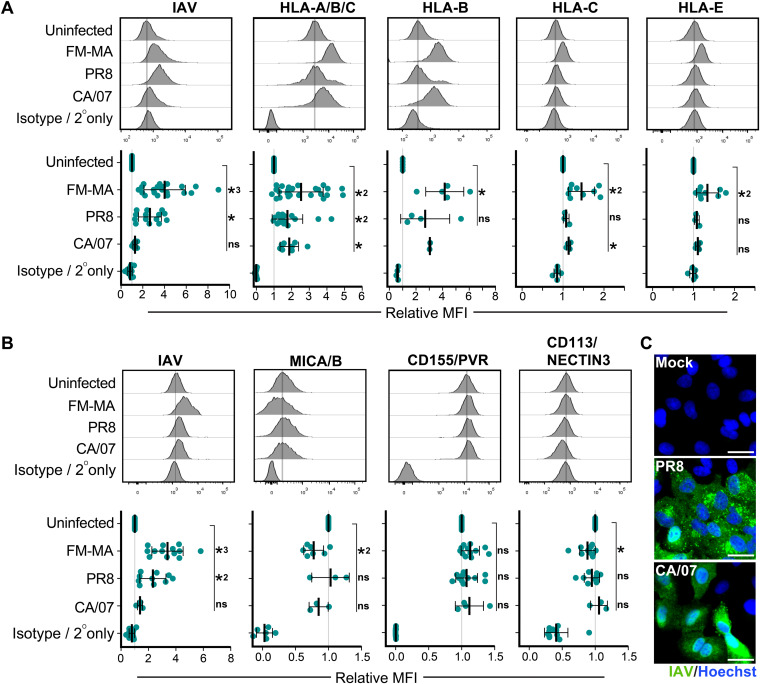
IAV infection alters cell surface expression of ligands for NK cell receptors. A549 cells were infected with FM-MA, PR8, or CA/07 at an MOI of 1. At 17 h, cells were fixed and immunostained to determine cell surface levels of NK cell ligands; cells were subsequently permeabilized for immunostaining of intracellular IAV proteins. (A) Flow cytometry analysis of cells immunostained with a pan-HLA-A/B/C antibody, or antibodies specific for class I HLA proteins HLA-B, HLA-C, or HLA-E or isotype antibody controls. (B) Flow cytometry analysis for cells immunostained with antibodies to detect NK cell-activating ligands MICA/B and CD155/PVR, or inhibitory ligand CD113/NECTIN3, or isotype antibody controls. Representative histograms (top) show results of a single experiment; the vertical lines represent the expression level of the target in uninfected cells. (Bottom) Mean fluorescence intensity (MFI) relative to uninfected cells. Each data point represents an independent experiment. Means ± SD are shown. *, *P* < 0.05; *2, *P* < 0.01; *3 *P* < 0.001. (C) A549 cells were mock infected or infected with PR8 for 16 h or CA/07 for 15 h at an MOI of 1, fixed, permeabilized, and stained with polyclonal antibodies specific for IAV proteins (green). DNA was stained with Hoechst-33342 (blue). Scale bar represents 20 μm.

To determine whether changes in NK cell ligand mRNA levels led to corresponding changes in the surface presentation of proteins, we infected A549 cells with PR8, FM-MA, or A/California/07/2009(H1N1) (CA/07) and analyzed cell surface expression of NK cell ligands by flow cytometry (Fig. 2). We observed a significant upregulation of HLA-A/B/C on the surface of PR8-, FM-MA-, and CA/07-infected cells (Fig. 2A), which correlated with our RT-qPCR data (Fig. 1B). When measured individually, HLA-B, -C, and -E were significantly upregulated by FM-MA infection, whereas PR8 and CA/07 infections elicited modest increases in HLA-B, which did not achieve statistical significance (Fig. 2A). A modest but statistically significant increase in HLA-C was observed in the CA/07 infections. MICA/B ligands for the activating NKG2D receptor were differentially regulated by infection; PR8 and CA/07 infections had no effect on cell surface levels of MICA/B, whereas FM-MA infection caused a modest downregulation (Fig. 2B) consistent with our RT-qPCR data (Fig. 1B). We also observed a significant downregulation of CD113/NECTIN3 in FM-MA-infected cells (Fig. 2B) consistent with our RT-qPCR data (Fig. 1B) and bioinformatics analysis data (Fig. 1A). Curiously, even though all infections were performed at an MOI of 1, immunostaining for IAV antigens with a polyclonal anti-IAV antibody was quite variable between IAV strains in our flow cytometric analysis (Fig. 2A and B); we performed additional immunofluorescence staining of adherent A549 cell monolayers to confirm efficient infection of these cells with PR8 and CA/07 viruses (Fig. 2C). Taken together, our bioinformatic analysis of published gene expression data sets, combined with our own RT-qPCR and surface staining experiments, clearly demonstrate that IAV infection alters the expression of NK cell ligands and that the most striking and consistent finding is increased surface presentation of class I HLA proteins, in agreement with previous studies (28, 29).

### Defective vRNAs increase surface HLA presentation in a MAVS-dependent manner.

**FIG 3:**
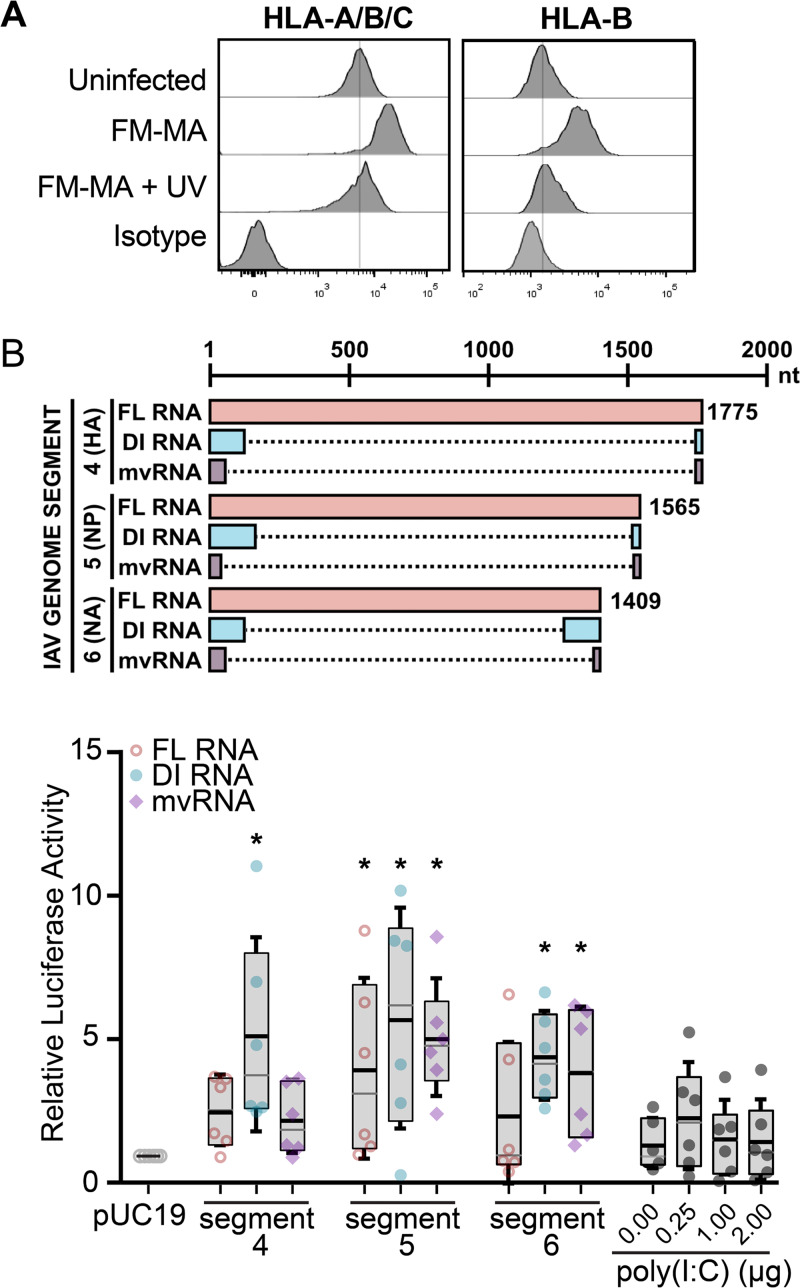
Defective viral RNAs increase ISRE-dependent luciferase activity in A549 cells. (A) FM-MA inoculum was exposed to UV light prior to infection of A549 cells at MOI of 1. At 17 hpi, cells were fixed and immunostained with a pan-anti-HLA-A/B/C antibody or an anti-HLA-B antibody and processed for flow cytometry. The vertical lines indicate the HLA expression level in uninfected cells. Representative data from one out of two independent experiments is shown. (B) (Top) Cartoon compares RNAs derived from the indicated genome segments and expressed from pPolI-based minireplicon plasmids, including full-length (FL) vRNA, defective interfering (DI) vRNA, or mini-viral RNA (mvRNA); dashed lines mark internal deletions on the DI RNAs and mvRNAs. (Bottom) A549 cells were transfected with IAV minireplicons expressing the indicated FL vRNAs, DI RNAs, or mvRNAs derived from the indicated genome segments. An ISRE-driven firefly luciferase reporter plasmid was cotransfected with minireplicon plasmids to measure IFN signaling, along with a Renilla luciferase plasmid that served as normalization control. Poly(I·C) and an empty pUC19 plasmid served as positive and negative controls, respectively. Firefly luciferase activity was normalized to Renilla luciferase control for each sample, and data were expressed as fold change compared with pUC19 plasmid transfection (*n* = 6; *, *P* < 0.05; IQR boxes and SD whiskers are shown).

To determine whether HLA upregulation was a consequence of IAV entry or later steps in viral replication, we infected A549 cells with UV-inactivated or control FM-MA virus and measured cell surface HLA levels by flow cytometry using a pan-HLA-A/B/C antibody or an HLA-B-specific antibody. UV treatment damages viral RNA and prevents transcription and replication of the viral genome (31). We observed that, unlike infectious virus that increased cell surface HLA as expected, UV-inactivated inoculum had no effect (Fig. 3A).

During replication, the IAV RdRp frequently generates defective RNA products, including DI RNAs (12) and smaller mvRNAs (14). Like intact full-length vRNAs, DI RNAs bind to the viral nucleoprotein (NP) and assemble into viral ribonucleoprotein (vRNP) structures that limit RIG-I binding (13). In contrast, mvRNAs do not bind to NP and are thought to be primary RIG-I agonists (14). One consequence of IFN signal transduction is increased cell surface HLA presentation (32). Because UV-inactivated IAV was unable to increase HLA surface presentation, we reasoned that increased HLA gene expression could be triggered by innate immune responses activated by defective RNAs produced during viral replication. To test the ability of defective RNAs to induce IFN signaling in our system, we used an IFN-β-responsive luciferase reporter driven by an interferon-stimulated response element (ISRE) promoter element. We observed that transfection of A549 cells with IAV minireplicons designed to express mvRNAs, DI RNAs, or full-length vRNAs substantially induced ISRE-luciferase reporter activity (Fig. 3B). Interestingly, DI RNAs from genome segment 4 strongly activated ISRE-luciferase activity, whereas full-length vRNA or mvRNAs from the same segment had a moderate effect. In contrast, all three RNA species derived from genome segment 5 activated the ISRE-luciferase reporter to a similar extent. Notably, the virus-derived RNA species all potentiated stronger ISRE-luciferase reporter activity than poly(I·C), a known inducer of type I IFN in transfected A549 cells. Thus, in the A549 cell line used extensively in this study, diverse viral RNA species can elicit IFN signaling.

**FIG 4:**
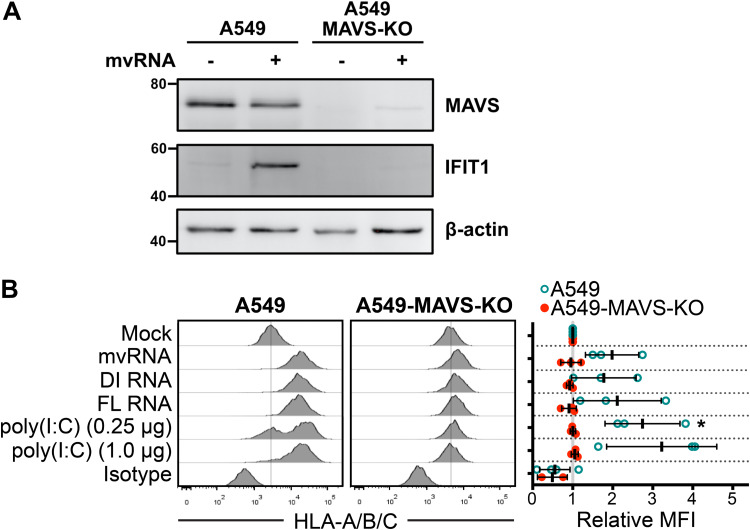
Defective viral RNAs increase surface HLA presentation in a MAVS-dependent manner. (A) A549 cells or A549-MAVS-KO cells were transfected with IAV minireplicon expressing mvRNA from genome segment 5 or empty pUC19 control for 24 h prior to harvest of protein lysates and immunoblotting with antibodies for the indicated target proteins. (B) A549 cells or A549 MAVS-KO cells were transfected with IAV minireplicon expressing defective vRNAs from genome segment 5 and analyzed by flow cytometry at 48 h posttransfection via surface immunostaining with a pan-anti-HLA-A/B/C antibody (*n* = 3). Histograms from a representative experiment are shown on the left; the vertical lines indicate the expression level of the target in uninfected cells. On the right, relative MFI values from at least 3 independent experiments are shown. *, *P* < 0.05.

Because many viruses selectively modulate HLA presentation to disrupt antiviral immune responses (33, 34), we wondered whether defective IAV vRNAs might affect HLA presentation. To test this directly, we transfected A549 cells with constructs encoding the IAV minireplicon system bearing mvRNA, DI RNA, or full-length vRNA species and measured surface levels of HLA. Control cells transfected with poly(I·C) showed a strong dose-dependent upregulation of surface HLA-A/B/C over a 48-h period (Fig. 4B). Cells expressing viral RNAs from segment 5 likewise displayed strong surface HLA staining, indicating that they are sufficient to increase cell surface HLA in the absence of infection, in agreement with their ability to stimulate the ISRE-luciferase reporter.

RIG-I binds to IAV RNA panhandle structures and assembles with the MAVS adaptor on the surface of mitochondria and peroxisomes to drive antiviral signal transduction and IFN production (9, 10). To determine whether MAVS was required for IFN-stimulated gene (ISG) production following the detection of defective viral RNAs in our system, we transfected A549 cells and MAVS-deficient A549-MAVS-knockout (KO) cells with an IAV minireplicon that expressed mvRNAs derived from genome segment 5. We observed a strong upregulation of interferon-induced protein with tetratricopeptide repeats 1 (IFIT1) in the mvRNA-expressing A549 cells, whereas no IFIT1 was observed in A549-MAVS-KO cells (Fig. 4A). To test whether the RIG-I/MAVS axis was involved in HLA upregulation in our system, we measured surface HLA-A/B/C expression in parallel in transfected MAVS-deficient A549 cells (A549-MAVS-KO). Class I HLA levels on the MAVS-KO cells were unaffected by transfection with mvRNA, DI RNA, or full-length vRNA constructs (Fig. 4B). This finding indicates that HLA upregulation in IAV-infected cells results from activation of the RIG-I/MAVS pathway that recognizes viral replication intermediates.

### Class I HLA upregulation in IAV-infected cells is MAVS dependent.

**FIG 5:**
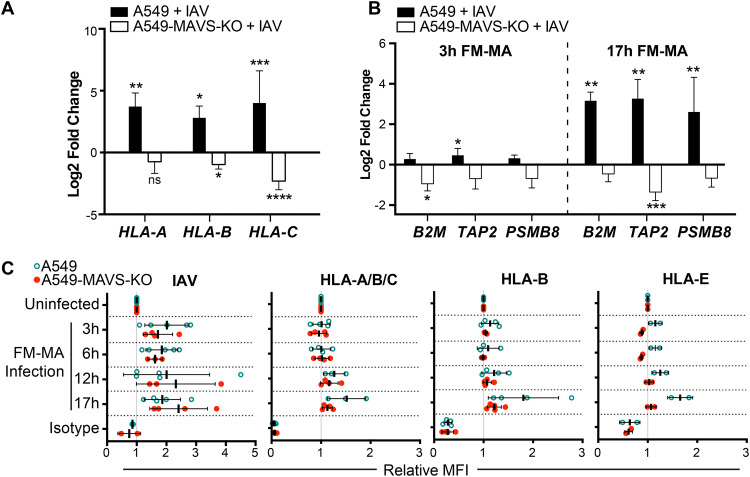
Class I HLA upregulation in IAV-infected cells is MAVS dependent. A549 cells or A549-MAVS-KO cells were infected with FM-MA at an MOI of 1. RNA was harvested for RT-qPCR at 3 hpi or 17 hpi. (A) Relative fold change in HLA-A, -B, and -C transcript levels in A549 cells or A549-MAVS-KO cells at 17 hpi (*n* = 3). (B) Relative fold change in B2M, TAP, and PSMB8 transcript levels in A549 and A549 MAVS-KO cells at 3 hpi or 17 hpi (*n* = 3). (C) Relative MFI of cell surface HLA proteins in FM-MA-infected A549 cells and A549-MAVS-KO cells at indicated times, relative to uninfected controls. *, *P* < 0.05; **, *P* < 0.01; ***, *P* < 0.001.

Having established that the RIG-I/MAVS axis is involved in HLA upregulation in response to ectopic expression of viral replication intermediates, we next addressed the role of RIG-I/MAVS in authentic IAV infection. A549 cells or A549-MAVS-KO cells were infected with FM-MA virus, and class I HLA expression was measured by flow cytometry. At 17 hours postinfection (hpi), class I HLA-A, -B, and -C mRNA levels were significantly increased in infected wild-type (WT) A549 cells compared with mock-infected control cells but did not increase in MAVS-KO cells (Fig. 5A). The expression of other components of the antigen processing and presentation machinery, including β2 microglobulin (B2M), transporter associated with antigen processing (TAP1) and proteasome subunit beta 8 (PSMB8) was significantly increased in A549 cells by 17 hpi but not in MAVS-deficient cells (Fig. 5B). Surface class I HLA levels were largely unchanged in the early stages of infection, with moderate increases first measured at 12 hpi and further increases by 17 hpi (Fig. 5C). Cell surface levels of the nonclassical HLA-E also increased on A549 cells over the infection time course. In contrast, cell surface levels of these classical and nonclassical HLA proteins remained unchanged in the A549-MAVS-KO cells throughout the time course, despite robust accumulation of viral proteins indicative of progression of the infectious cycle (Fig. 5C). Together, these findings indicate that MAVS is required for IAV-induced HLA upregulation on the surface of infected A549 cells.

### Defective IAV RNAs elicit cell-intrinsic and paracrine upregulation of class I HLA proteins.

**FIG 6:**
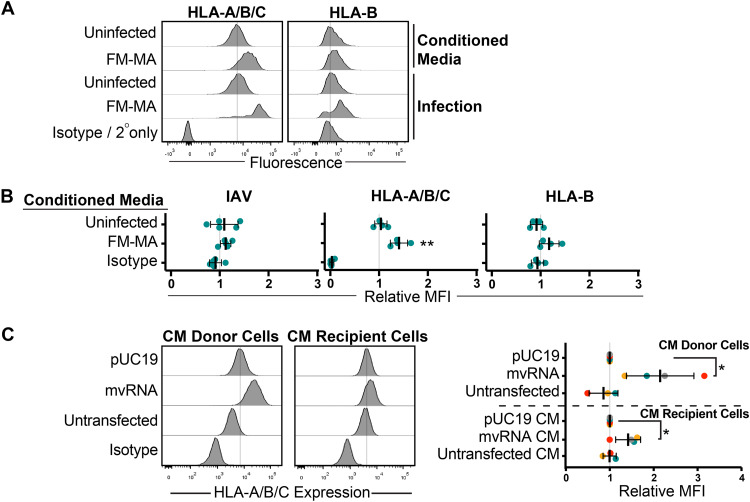
Defective IAV RNAs elicit cell-intrinsic and paracrine upregulation of class I HLA proteins. (A) A549 cells were treated with conditioned medium containing UV-inactivated culture supernatant from FM-MA-infected cells. Surface HLA levels on recipient cells (17 h posttreatment) and infected donor cells (17 hpi) were determined by flow cytometry. Histograms from a representative experiment are shown. Vertical dashed-lines indicate the expression level in uninfected cells. (B) MFI of cell surface HLA proteins on recipient cells from A relative to cells treated with conditioned media from mock-infected cells. Each data point represents an independent experiment. (C) A549 cells were treated with conditioned medium from cells transfected with IAV minireplicon expressing defective vRNAs from genome segment 5 or from control untransfected cells or pUC19 vector-transfected cells. After 24 h, cells were fixed and immunostained with a pan-anti-HLA-A/B/C antibody (*n* = 3). Histograms from a representative experiment are shown on the left; vertical lines indicate the expression level of targets in uninfected cells. On the right, relative MFI values from at least 3 independent experiments are shown (*, *P* < 0.05).

Because signaling downstream of type I IFN receptors increases class I HLA expression (32), we investigated the contribution of soluble factors to HLA expression in IAV-infected cells. We infected A549 cells with FM-MA for 17 h and collected cell supernatants, which were UV treated to inactivate virions prior to incubation with naive A549 cells for an additional 17 h. Donor and recipient A549 cells were stained with anti-HLA-A/B/C or anti-HLA-B antiserum and analyzed by flow cytometry. We observed marked increases in surface class I HLA proteins on IAV-infected A549 cells as before, compared with moderate increases on cells incubated with UV-treated conditioned medium (Fig. 6A and B). Staining cells with anti-IAV antiserum confirmed that the UV treatment of culture supernatants inactivated virions and prevented subsequent infection of naive A549 cells, mitigating concerns of residual infection in these experiments (Fig. 6B). Incubating naive A549 cells with culture supernatants collected from cells expressing IAV mvRNAs yielded a similar result, with strong significant increases in class I HLA protein levels on the donor cells compared with relatively modest increases on the cells that received the conditioned medium (Fig. 6C). Together, these findings indicate that class I HLA can indeed be upregulated on epithelial cells in a paracrine manner in response to infection, but this effect is weaker than the cell-intrinsic class I HLA upregulation on the infected cell.

### HLA upregulation in response to defective IAV RNAs is dependent on IFN signaling.

**FIG 7:**
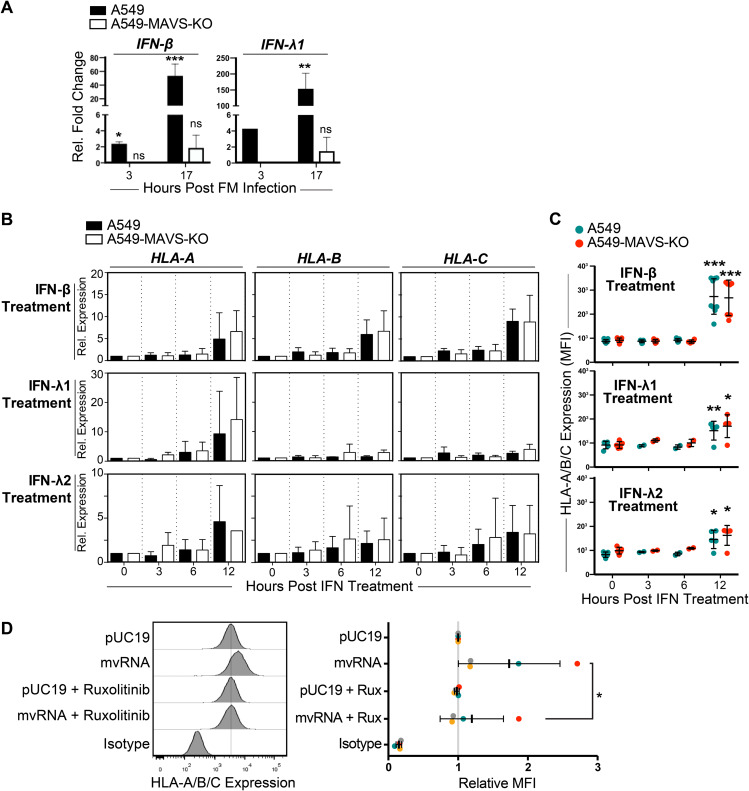
HLA upregulation in response to defective IAV RNAs is dependent on IFN signaling. (A) A549 cells or A549-MAVS-KO cells were infected with FM-MA for 17 h, and relative levels of IFN-β and IFN-λ1 transcripts compared with uninfected controls were analyzed by RT-qPCR (*n* = 3). (B) A549 cells or A549-MAVS-KO cells were treated with recombinant IFN-β, IFN-λ1, or IFN-λ2, and RNA was harvested over a 12-h time course. The relative expression of HLA-A, -B, and –C transcripts was analyzed by RT-qPCR. (C) The surface expression of HLA-A/B/C was determined by immunostaining and flow cytometry of cells harvested over the time course of IFN treatment described in B (*n* = 3). (D) Analysis of HLA surface expression on A549 cells transfected with IAV minireplicon expressing defective vRNAs from genome segment 5 or from control pUC19 vector-transfected cells. At 6 h posttransfection, cells were treated with ruxolitinib (Rux) or mock treated. *, *P* < 0.05; **, *P* < 0.01; ***, *P* < 0.001.

IAV infection induces production of type I and type III IFN proteins by the infected cell that orchestrate autocrine and paracrine antiviral responses (1, 5). Compared with uninfected A549 cells, infection with FM-MA induced MAVS-dependent expression of *IFN-β* and *IFN-λ1* genes as early as 3 h postinfection, which increased to 50-fold and 150-fold, respectively, by 17 h postinfection (Fig. 7A). To confirm that type I and type III IFNs can induce HLA upregulation in our system, we treated A549 cells with IFN-β, IFN-λ1, or IFN-λ2 and compared HLA expression in IFN-treated and untreated cells. Both RT-qPCR and flow cytometry analysis showed that IFN-β was the most potent inducer of class I HLA mRNA and protein expression in A549 cells; HLA-A, HLA-B, and HLA-C mRNAs accumulated in IFN-β-treated cells by 12 h posttreatment (Fig. 7B), which was reflected in increased HLA-A/B/C cell surface staining (Fig. 7C). In contrast, 12-h treatment with IFN-λ1 potently increased HLA-A mRNA levels but not HLA-B and -C mRNA levels (Fig. 7B). Overall, IFN-β was a much more potent inducer of HLA in our system than IFN-λ1 and IFN-λ2.

Autocrine and paracrine type I and III IFN signaling is mediated by IFN receptor signal transduction via downstream nonreceptor tyrosine kinases Jak1, Jak2 and Tyk2 (35–39). To test if IFN receptor signaling plays a role in HLA upregulation, we treated A549 cells with the Jak1/Jak2 inhibitor Rux. In A549 cells transfected with the mvRNA-expressing IAV minireplicon, we observed that upregulation of HLA-A/B/C on the cell surface was inhibited by Rux treatment (Fig. 7D). In control pUC19-transfected cells, Rux had no effect on HLA levels. These data suggest that signaling downstream of IFN receptors through Jak1 and/or Jak2 could play a role in HLA upregulation triggered by IAV replication intermediates. However, because signaling downstream of many type I cytokine receptors also involves Jak1 and/or Jak2, the contribution of these cytokine receptors cannot be ruled out in this experiment (40–42).

### NS1 protein limits cell-intrinsic and paracrine upregulation of class I HLA proteins.

**FIG 8:**
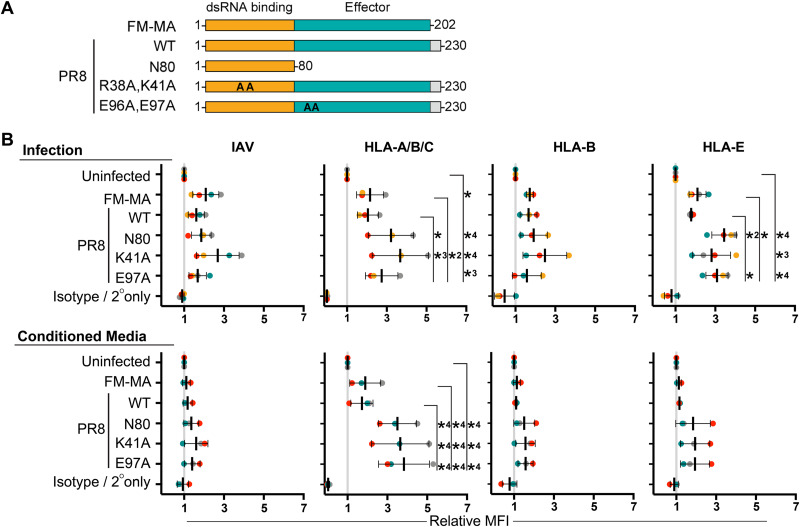
NS1 protein limits cell-intrinsic and paracrine upregulation of class I HLA proteins. (A) A cartoon representing wild-type and mutant NS1 proteins used in this study. A carboxy-terminal disordered tail region present in PR8 NS1 and absent in FM-MA NS1 is shown in gray. Positions of alanine substitutions in R38A, K41A and E96A, E97A mutant proteins are indicated as “AA.” Amino-terminal double-stranded RNA (dsRNA) binding domain is in orange; effector domain is in teal. (B) A549 cells were infected with the indicated viruses at an MOI of 1 or mock infected. At 17 hpi, cell supernatants were collected prior to cell fixation and transferred to naive A549 cells for an additional 17 h of incubation prior to fixation. Donor and recipient cells were immunostained with the indicated anti-HLA antibodies to determine cell surface levels of NK cell ligands; cells were subsequently permeabilized for immunostaining of intracellular IAV proteins and analyzed by flow cytometry. (Top) Data from donor-infected or mock-infected cells. (Bottom) Data from cells exposed to conditioned media. Data are presented as MFI relative to uninfected cells or conditioned media treatment from uninfected cells. Each data point represents an independent experiment. Means ± SD are shown. *, *P* < 0.05; *2, *P* < 0.01; *3, *P* < 0.001; *4, *P* < 0.0001 indicate significance determined by Tukey’s multiple-comparison test.

In many experiments, FM-MA infections elicited larger increases in class I HLA levels than PR8 infections. IAV genome segment 8 encodes the primary innate immune antagonist protein nonstructural protein 1 (NS1), which is highly variable between strains. The FM-MA NS1 protein lacks the carboxy-terminal 28 amino acids found in PR8 NS1 (Fig. 8A). To assess the role of NS1 in HLA upregulation, we infected A549 cells with FM-MA and PR8, as well as a panel of PR8 viruses with NS1 mutations that compromise its ability to suppress innate immune responses. These include point mutations in NS1 that disrupt its ability to suppress RIG-I activation (R38A, K41A or E96A, and E97A) (43, 44) and a larger deletion that removes the effector domain and disordered carboxy-terminal tail (N80) (45). Consistent with known properties of NS1 in suppressing IFN production, all three NS1-mutant viruses caused HLA upregulation, and this upregulation was higher than the parental PR8 strain or the FM-MA strain (Fig. 8B, top). Incubation of A549 cells with UV-inactivated culture supernatants from these infections revealed a key role for NS1 in limiting paracrine signaling. Conditioned media from FM-MA or PR8 infections caused moderate increases in HLA-A/B/C levels, whereas media from NS1 mutant virus infections elicited striking, statistically significant increases in HLA-A/B/C and showed a trend toward increased HLA-B and HLA-E levels when measured independently (Fig. 8B, bottom). Together, these findings clearly demonstrate that NS1 plays a lead role in suppressing the HLA presentation in IAV-infected cells and bystander cells alike.

## DISCUSSION

NK cell receptors bind to ligands on the surface of infected cells and initiate antiviral immune responses by integrating activating and inhibitory signals. Here, we show that IAV infection of cultured epithelial cells alters the expression of an array of ligands for activating and inhibitory NK cell receptors. With some exceptions, we observed a general trend toward increased expression of ligands for inhibitory receptors and downregulation of ligands for activating receptors, suggesting that the net effect of viral reprogramming of epithelial cells could be the suppression of NK cell responses. Class I HLA proteins are recognized by KIR proteins on NK cells, and increased binding of KIR by HLA on IAV-infected cells *in vitro* has been shown to inhibit NK cell activity (28). However, the mechanisms that control HLA upregulation on IAV-infected cells are not fully understood. Here, we report that class I HLA upregulation depends on postentry steps in replication because UV-inactivated virus had no effect on HLA gene expression or accumulation of HLA proteins on the surface of A549 epithelial cells. We observed that defective viral RNAs produced from an IAV minireplicon were sufficient to induce expression and cell surface presentation of class I HLA. Knowing that the RIG-I/MAVS signaling axis is the primary mechanism of detection of IAV replication intermediates in infected cells that drives antiviral responses, we tested HLA upregulation in MAVS-deficient cells. We observed that genetic deletion of MAVS prevented class I HLA upregulation in response to IAV infection or ectopic expression of mvRNAs or DI RNAs, suggesting that aberrant viral RNAs generated during infection are bound by RIG-I and transduce signals that increase HLA gene expression.

mvRNAs are potent inducers of RIG-I-mediated type I IFN production, whereas DI RNA and full-length vRNA are bound by the NP, which limits their recognition by RIG-I (14). Our minireplicon assays showed an induction of ISRE-luciferase activity and MAVS-dependent class I HLA upregulation by all three RNA species, but we cannot rule out the possibility that mvRNAs generated from the longer constructs could contribute to these outcomes. Alternatively, it is possible that the minireplicon constructs did not express sufficient NPs in transfected A549 cells to cloak the DI RNA and FL-vRNA products, resulting in RIG-I binding, IFN production, and class I HLA upregulation. Future studies must focus on manipulating the levels of different defective RNA species produced during infection to overcome the limitations of IAV minireplicon experiments and more firmly establish the mechanism of action for mvRNAs and other defective viral RNAs.

Our work shows that IAV infection causes MAVS-dependent increases in the expression of antigen processing and presentation machinery, including class I HLA-A, -B and -C and associated B2M, TAP1, and PSMB8 proteins, as well as the nonclassical HLA-E protein. They comprise an antiviral gene expression program that responds to the detection of defective viral RNAs by RIG-I. Cytotoxic T cells (CTLs) and NK cells rely on HLA proteins for target cell recognition (46–48). Specifically, CTL activation and lysis of target cells require binding to class I HLA proteins loaded with viral peptide antigens or HLA-E proteins loaded with noncanonical peptides from viruses and stress-related proteins (47, 49). In contrast, NK cell activation is inhibited by increased HLA protein levels on the surface of virus-infected cells by engaging inhibitory KIR proteins, as a main function of NK cells is to destroy host cells that have no surface expression of class I HLA proteins (28, 33, 34). Our observations are consistent with numerous reports of viruses that induce class I HLA expression or encode structurally similar immunoevasins that engage inhibitory receptors on NK cells and undermine their activity (29, 33, 34, 50–53). Thus, MAVS-dependent increases in cell surface class I HLA proteins could skew antiviral immune responses to thwart NK cells at the expense of potential CTL activation. This suggests that NK cells represent an existential threat for many viruses.

In the course of these studies, we discovered that IFN can amplify responses to aberrant viral RNA products to increase HLA presentation. Specifically, we found that IAV infection of A549 cells stimulated the production of IFN-β and IFN-λ1 in a MAVS-dependent manner, which dramatically increased at later times postinfection. Conditioned medium collected from these infected cells elicited modest, but significant, increases in HLA presentation on naive epithelial cells that paled in comparison to the magnitude of increase on the donor infected cells. Conditioned medium collected from cells expressing IAV mvRNAs and DI RNAs similarly induced modest increases in surface class I HLA proteins when incubated with naive A549 cells. We have not yet taken steps to fully characterize the composition of these culture supernatants, but the available evidence points to a role for type I IFNs and, to a lesser extent, type III IFNs. However, it remains formally possible that additional factors secreted by infected cells could contribute to HLA gene expression.

HLA is upregulated in response to infection by a wide array of viruses, but underlying mechanisms differ. Hepatitis C virus (HCV) infection indirectly increases cell surface class I HLA levels by increasing the expression of TAP1 and aiding transport of processed peptides to the endoplasmic reticulum where they can be loaded onto HLA and transported to the cell surface (34). Similarly, West Nile virus (WNV) infection increases TAP1 activity, resulting in the increased transport of processed peptide antigens into the endoplasmic reticulum (ER) and higher levels of HLA:peptide complexes on the surface of infected cells (54). In contrast, Zika virus infection stimulates the RIG-I/MAVS/IRF3 pathway and downstream IFN-β expression, which increases HLA expression in infected cells (33). This mechanism is quite similar to the one we describe herein for IAV, except that in Zika virus-infected cells, RIG-I binds to the 5′-triphosphate end of the intact (+)-sense single-stranded RNA (ssRNA) virus genome (55) rather than defective RNA products of the IAV polymerase.

NS1 normally prevents RIG-I-mediated detection of defective viral RNAs and downstream IFN signal transduction. Here, we demonstrated that NS1 restrained class I HLA presentation on infected cells. It also had a dramatic impact on HLA expression in bystander cells; treatment of naive A549 cells with UV-inactivated culture supernatants collected from NS1 mutant virus infections elicited strong class I HLA upregulation compared with controls. However, NS1 has also been shown to increase the transcription of the endoplasmic reticulum aminopeptidase 1 (*ERAP1*), which encodes a component of the antigen presentation machinery (56). Thus, the effect of NS1 on class I HLA-mediated antigen presentation is not limited to IFN inhibition. More detailed studies of this hypervariable virulence factor will be required to fully understand the impact of NS1 on innate immune responses involving NK cells.

The existence of aberrant IAV RNA species has been well documented, but it has been less clear whether these products can benefit the virus. There is substantial evidence that defective RNA products of the viral polymerase limit productive viral replication by inducing IFN responses and promoting the generation of defective viral particles when incorporated into viral progeny. Our work demonstrates that aberrant IAV mvRNAs and DI RNAs stimulate class I HLA expression, which may aid the viral evasion of NK cell-mediated immune responses.

## MATERIALS AND METHODS

### Gene expression analysis.

We retrieved influenza A virus infection gene expression data sets from the GEO database that met the following inclusion criteria: infection of human alveolar adenocarcinoma A549 cells or primary human lung cells with matched mock-infected controls (see Table S1 for accession numbers, cell lines, and viruses). We manually curated a gene set encoding known NK cell ligands. NK ligand genes were divided into activating or inhibitory ligands and a third group with ambiguous or context-dependent function. Class I HLA genes were highlighted as a separate group due to their prominent role in controlling NK cell activation. Data were analyzed with the NCBI GEO2R tool (https://www.ncbi.nlm.nih.gov/geo/geo2r/) using the profile graph feature to obtain relative expression values, which were then processed using the equation below to obtain the fold expression change, followed by log_2_ transformation to aid data visualization (57, 58). Data were plotted as median values with interquartile range (IQR) shown. Positive values indicated a relative increase in NK ligand gene expression in infected cells compared to mock-infected controls, whereas negative values indicated the opposite.

$$\text{relative fold change = }\frac{\text{mean}\left(\left(\frac{\text{(raw infected value 1)}}{\text{(median uninfected values 1)}}\right)\text{, …,}\left(\frac{\text{(raw infected value n)}}{\text{(median uninfected values n)}}\right)\right)}{\text{mean}\left(\left(\frac{\text{(raw infected value 1)}}{\text{(median uninfected values 1)}}\right)\text{, …,}\left(\frac{\text{(raw infected value n)}}{\text{(median uninfected values n)}}\right)\right)}$$

### Cell lines.

A549 cells and derivatives were cultured in complete Dulbecco’s modified Eagle’s medium (DMEM) supplemented with 10% fetal bovine serum (Thermo Fisher Scientific) at 37°C and 5% CO_2_. To generate A549-MAVS-KO cells, A549 cells were seeded at 1.65 × 10^5^ cells per well in a 12-well cluster dish to obtain a confluence of 80% the next day. One hour before transfection, the medium was changed to F12 medium supplemented with 1% l-glutamine and 10% Fetalclone III serum (FCl-III) (Thermo Fisher Scientific). A total of 1.6 μg of Cas9 nuclease expression plasmid (number U-005200-120; Dharmacon), 50 nM *trans*-activating CRISPR RNA (tracrRNA; Dharmacon), and 50 nM crRNA (crRNA nontargeting control 1 number U-007501-05 or crRNA human MAVS [gene ID: 57506] ex2, number GRANB-000259) were transfected with 40 μg/ml Dharmafect DUO transfection reagent (Dharmacon). A total of 48 h later, 2 μg/ml puromycin (Sigma-Aldrich) was added to select for cells that integrated the Cas9 expression plasmid. Cells were cultured for 7 days. Monoclonal populations were obtained by seeding cells at 40 cells/ml, and clones were isolated using cloning rings. Gene editing was confirmed by Sanger sequencing at the Génome Québec Innovation Centre (McGill University, Montréal, Quebec, canada). The CRISP-ID Web application tool (59) was used to locate the targeted region and monitor the insertions/deletions within the gene. crRNA nontargeting control sequence was GATACGTCGGTACCGGACCG. The crRNA human MAVS (gene ID: 57506) ex2 sequence was GGATTGGTGAGCGCATTAGA.

### Viruses and infections.

Wild-type (WT) influenza A/Puerto Rico/8/1934(H1N1) (PR8) virus was generated using the 8-plasmid reverse genetic system (60) as previously described (40, 42). Viral stocks were produced in Vero cells, and titers were determined by plaque assays in Vero cells. NS1 mutations were verified by Sanger sequencing of virus stocks. The mouse-adapted influenza A/Fort Monmouth/1/1947 (FM-MA) virus was a generous gift from Earl G. Brown (University of Ottawa, Ottawa, Canada). Influenza A/California/07/2009 was acquired from Todd Hatchette (Dalhousie University, Halifax, Canada). Viral stocks were produced in MDCK cells, and infectious titers were determined by plaque assays in MDCK cells. All plaque assays were performed using 1.2% Avicel overlays as described in Matrosovich et al. (61). Plaque assays and virus production in MDCK cells were performed in the presence of 1 μg/ml tosyl phenylalanyl chloromethyl ketone (TPCK)-treated trypsin (Sigma-Aldrich), whereas similar procedures in Vero cells employed 2.5 μg/ml TPCK-treated trypsin. A549 cell monolayers were mock infected or infected with the WT or mutant viruses at an MOI of 1 for 1 h at 37°C. Monolayers were washed with phosphate-buffered saline (PBS) and overlaid with fresh infection media (0.5% bovine serum albumin [BSA] in DMEM supplemented with 20 μM l-glutamine) and incubated at 37°C in 5% CO_2_ atmosphere.

### Plasmids, transfections, and luciferase assays.

Minireplicon plasmids expressing full-length (FL) vRNA, DI RNA, and mvRNA under the control of a RNA polymerase I (pol I) promoter were a generous gift from Aartjan te Velthuis (Cambridge University, Cambridge, UK). A549 cells were cotransfected using Lipofectamine 2000 (Thermo Fisher Scientific) with plasmids encoding the three polymerase subunits (PB1, PB2, and PA) and NP from A/Udorn/307/1972(H3N2) IAV, a generous gift from Yoshihiro Kawaoka (University of Wisconsin-Madison), and luciferase reporter plasmids under the control of the interferon-stimulated response element (ISRE) promoter (firefly) and the CMV promoter (Renilla). At 24 h posttransfection, cells were washed with PBS and lysed in 1× reporter lysis buffer (Promega). The dual-luciferase assay was performed at 24 h posttransfection using the Dual-Glo luciferase assay system (Promega). Jak1/2 was inhibited with 5 μM INCB018424, also known as ruxolitinib (Rux) (Cedarlane, Burlington, Canada); A549 cells were transfected with IAV minireplicons expressing mvRNAs from genome segment 5 or no mvRNA control (pUC19 plasmid). At 6 h posttransfection, cells were treated with Rux or mock-treated and were harvested 24 h posttransfection for flow cytometry.

### RNA purification, cDNA preparation, and qPCR.

RNA was extracted from cells using the TRIzol reagent (Thermo Fisher Scientific) and a Quick-RNA miniprep kit (Zymo Research). In all cases, RNA was treated with Turbo DNase (Thermo Fisher Scientific) prior to reverse transcription using a Verso cDNA synthesis kit (Thermo Fisher Scientific). qPCR was performed using iTaq universal SYBR green supermix (Bio-Rad) on a Bio-Rad CFX Connect instrument and analyzed using the Bio-Rad CFX Manager 3.1 program. Primers used are listed in Table 1.

**TABLE 1:** Primer sequences for RT-qPCR analysis

RT-qPCR target	Primer sequences (5′–3′)	Source
HLA-A	F-CGACGCCGCGAGCCAGA, R-GCGATGTAATCCTTGCCGTCGTAG	63
HLA-B	F-GACGGCAAGGATTACATCGCCCTGAA, R-CACGGGCCGCCTCCCACT	63
HLA-C	F-GGAGACACAGAAGTACAAGCG, R-CGTCGTAGGCGTACTGGTCATA	63
HLA-E	F-CCTACGACGGCAAGGA, R-CCCTTCTCCAGGTATTTGTG	63
MIC-A	F-ACTTGACAGGGAACGGAAAGGA, R-CCATCGTAGTAGAAATGCTGGGA	63
MIC-B	F-ATCTGTGCAGTCAGGGTTTCTC, R-TGAGGTCTTGCCCATTCTCTGT	63
PVR	F-GCTCTGCTGTTTGTTCTGCTTTCC, R-TTTCTGCTGCTGGATGCGGTTT	63
NECTIN1	F-ACTACCACATGGACCGCTTC, R-GTTGATGGGTCCCTTGAAGA	Designed in-house
NECTIN2	F-TGGACTGGGAAGCCAAAGAGA, R-TACAGAGAGGGTCACAGGTATCAGG	63
NECTIN3	F-GTTACATTCCCGCTTGGAAA, R-CCCAGTCAATATGTGCAACG	Designed in-house
CADM1	F-GGTGGAAGAGTGGTCAGACA, R-CTTCCCGATGGCTTCACATG	Designed in-house
CDH1	F-AGGAATCCAAAGCCTCAGGT, R-ACCCACCTCTAAGGCCATCT	Designed in-house
CDH2	F-GAGGCAGAGACTTGCGAAAC, R-CCATTAAGCCGAGTGATGGT	Designed in-house
PCNA	F-CGGATACCTTGGCGCTAGTA, R-CACTCCGTCTTTTGCACAGG	Designed in-house
CD27L	F-TGGTACACATCCAGGTGACG, R-AGGCAATGGTACAACCTTGG	Designed in-house
IFN-β	F-GTCTCCTCCA AATTGCTCTC, R-ACAGGAGCTTCTGACACTGA	64
IFN-λ1 (IL-29)	F-GAAGCAGTTGCGATTTAGCC, R-GAAGCTCGCTAGCTCCTGTG	65
IFN-λ2 (IL-28A)	F-GCCAAAGATGCCTTAGAAGAG, R-CAGAACCTTCAGCGTCAGG	66
5S rRNA	F-GCCCGATCTCGTCTGATCT, R-AGCCTACAGCACCCGGTAT	Designed in-house
GAPDH	F-ACGAATTTGGCTACAGCAACAGGG, R-TCTACATGGCAACTGTGAGGAGG	37

### Flow cytometry.

IAV-infected (3 to 17 h), mock-infected, or transfected A549 or A549-MAVS-KO cells were harvested, washed with FACS buffer containing 0.5% BSA and 0.02% sodium azide in phosphate-buffered saline (PBS), and stained with APC-conjugated anti-HLA-A/B/C antibodies (clone W6/32; BioLegend), FITC-conjugated anti-HLA-C antibodies (catalog [cat.] number NBP2-50419F; Novus Biologicals), PerCP/Cyanine5.5-conjugated anti-HLA-E antibodies (cat. number 342609; BioLegend), PE/Cy7-conjugated anti-CD155/PVR antibodies (cat. number 337613; BioLegend), FITC-conjugated anti-CD113/NECTIN3 antibodies (cat. number sc-69715; Santa Cruz Biotechnology), or PE/Cy7-conjugated anti-MICA/B antibodies (cat. number 320917; BioLegend) in FACS buffer at 4°C for 20 min. Cells stained with unconjugated primary mouse antibodies against HLA-B (cat. number NBP-245001; Novus Biologicals) were subsequently stained with FITC-conjugated rat anti-mouse secondary antibody (cat. number 11-4011-85; eBioscience). For detection of IAV proteins, cells were fixed and permeabilized using fixation/permeabilization buffers (BioLegend) and stained with anti-IAV polyclonal antibody (cat. number ab20841; Abcam) and phycoerythrin (PE)-conjugated secondary antibody (cat. number sc-3743; Santa Cruz Biotechnology). All cells were counterstained with a viability dye (cat. number 65-0865-14; eBioscience fixable viability dye eFluor 780). Transfected cells were fixed in 1% paraformaldehyde without permeabilization and intracellular staining. After a final wash in FACS buffer, cells were analyzed on a BD LSRFortessa FACS analyzer. Data were analyzed with FlowJo X software (Ashland, OR).

### Immunofluorescence microscopy.

For immunofluorescence microscopy, A549 cells were seeded on glass coverslips and cultured overnight prior to PR8 or CA/07 infection at an MOI of 1 or mock infection. At the indicated times postinfection, cells were fixed with 4% paraformaldehyde and permeabilized with cold methanol, as described in reference 62. IAV antigens were detected by incubation with goat polyclonal anti-influenza A virus antibody (ab20841; Abcam Inc., Toronto, ON, Canada) at 1:400 dilution, followed by Alexa-488-coupled donkey anti-goat secondary antibody (Thermo Fisher Scientific) at 1:1000 dilution, along with 5 ng/ml Hoechst dye (blue). Images were captured using a Zeiss AxioImager Z2 microscope. Scale bars represents 20 μm.

### Western blots.

A549 cells or A549-MAVS-KO cell monolayers were transfected with IAV minireplicons expressing mvRNA from genome segment 5 or empty pUC19 plasmid control for 24 h. Cell monolayers were washed once with ice-cold PBS and lysed in 2× Laemmli buffer (4% [wt/vol] sodium dodecyl sulfate [SDS], 20% [vol/vol] glycerol, and 120 mM Tris-HCl [pH 6.8]). DNA was sheared by repeated pipetting with a fine-gauge needle before 100 mM dithiothreitol (DTT) addition and boiling at 95°C for 5 min. Samples were stored at –20°C until analysis. Total protein concentration was determined by DC protein assay (Bio-Rad), and equal quantities were loaded in each SDS-PAGE gel. Proteins were transferred to polyvinylidene difluoride (PVDF) membranes (Bio-Rad) with the Trans-Blot Turbo transfer apparatus (Bio-Rad). Membranes were blocked with 5% bovine serum albumin Tris-buffered saline with Tween 20 (TBST; 0.1% [vol/vol] Tween) before probing overnight at 4°C with antibodies raised to the following targets: MAVS (Cell Signaling Technologies [CST] number 24930, D5A9E), IFIT1 (CST number 14769, D2X9Z), and β-actin (CST number 5125). Membranes were washed with TBST and incubated with goat anti-rabbit-HRP antibody (CST number 7074) prior to detection with the Clarity-ECL chemiluminescence reagent (Bio-Rad). All blots were imaged on a Bio-Rad ChemiDoc-Touch system. Molecular weights were determined using protein standards (number P7719; New England BioLabs).

### Culture supernatant transfer experiments.

Media from IAV-infected, mock-infected, or transfected A549 cells were collected and exposed to 1,200 J/m^2^ UV light in a HL-2000 HybriLinker (Mandel Scientific Company Inc., Guelph, Ontario, Canada) to inactivate the virus. Naive A549 cells were treated with culture supernatants for 17 h, and cells were analyzed by flow cytometry as described above.

### Interferon treatment experiments.

A549 and A549-MAVS-KO cells were plated in a 24-well plate (1 × 10^5^ cells/well) and were incubated for 24 h to achieve 80% confluence. Uninfected A549 cells and A549-MAVS-KO cells were treated with 100 units/ml IFN-β (PeproTech) or 100 ng/ml of IFN-λ1 or IFN-λ2 (PeproTech) in infection media (0.5% BSA DMEM + l-glutamine) or mock-treated and incubated at 37°C in 5% CO_2_ incubator for the indicated times prior to harvesting. Treated cells were then stained for flow cytometry or harvested for RNA extraction for RT-qPCR analysis.

### Statistical analyses.

We detected statistically significant events in the data without *a priori* sample size calculations, and therefore, no statistical methods were used to determine sample size. Statistical significance in comparisons among multiple groups of transcript levels for analyses of RT-qPCR and protein expression analyses by flow cytometry were performed using a one-way analysis of variance (ANOVA) test followed by Šidák’s *post hoc* test unless otherwise stated. A Tukey’s multiple comparisons posttest was used in Fig. 8b. All alpha levels were 0.05, with *P* values of <0.05 considered a significant difference. Statistical analyses were conducted using Prism 6.0 (GraphPad Software).
